# On Constipation

**Published:** 1854-09

**Authors:** 


*On Constipation.* By PROFESSOR TROUSSEAU.—The treatment should above all be directed to the removal of causes. It must not be thought that constipation is cured when an evacuation is procured; this would be as false as to imagine that a person is cured of wakefulness when he obtains sleep by opiates. We should seek not only to obtain relief for the moment, but regularity in the future. Constipation is not to be cured by Seidlitz powders or other purgatives, which, in the end, only increase it.

In the first place it is necessary to regulate the function; we should do everything to subject it to the empire of the will. We know how great is the power of habit over organic functions. When once accustomed to operate in a certain way, at certain hours, they have a strong tendency to continue to act thus. The habit of going to the privy is like the habit of urinating at certain periods, the habit of shaving, and of washing the face; when once these habits are established, they cannot be dispensed with. A constipated person should visit the water-closet at a convenient hour, he should remain for sometime, concentrating all his attention, all his will, all his contractile powers, on the act in question; he should make reiterated and energetic efforts to accomplish it. Whatever the result, he should return at the same hour on the morrow, and if, in the interval, a desire to go to stool is felt, it should be resisted, and the closet should be visited only at the hour chosen.

The diet should be altered. Instead of the abstemious diet often adopted by patients, they should be ordered abundant and nutritious food, of a kind which will at the same time promote digestion and leave a voluminous residue which will force the intestine to disembarrass itself. This disease, we have said, causes numerous disorders. Constipated persons always have oppression of the stomach and headache, for which they are ordered a light diet, white meats in small quantity, little wine, etc. This is an error. They should be desired to eat largely of roast meats. They should be compelled to eat, and reminded that, according to the English expression, one leg of mutton follows another.

But sometimes it is necessary to assist the will by an adjuvant, to facilitate an act, the habit of which is lost, as it were. We must beware of purgatives, but yet some medicine is necessary to assist defecation. Belladonna is this remedy *par excellence.* It does not purge; it only facilitates alvine evacuation. To what does it owe this property? It is difficult to say. Perhaps, as opium excites the cutaneous secretion and digitalis that of the urine, so belladonna may influence the liver, pancreas, or other glands opening on the intestinal tract. What is certain, however, is that the fifth of a grain of belladonna almost always procures several alvine evacuations. The dose may be increased to one grain, but as soon as the habit of defecation is restored, the dose of belladonna must be diminished and gradually abandoned.

Suppositories are much used, and commonly succeed well. Country people employ the middle stalk of the cabbage; physicians make them of hardened honey or cocoa.

Lastly, if constipation is connected with flaccidity of the abdominal walls, we must have recourse to a tight abdominal bandage, which will confine the intestines normally, and enable them to contract.—*Gazette des Hopitaux, from Virginia Med. and Surg. Jour.* O.

